# Surface Chemistry during Atomic Layer Deposition of Pt Studied with Vibrational Sum-Frequency Generation

**DOI:** 10.1021/acs.jpcc.1c06947

**Published:** 2022-01-31

**Authors:** V. Vandalon, A.J.M. Mackus, W.M.M. Kessels

**Affiliations:** Department of Applied Physics, Eindhoven University of Technology, 5600MB Eindhoven, The Netherlands

## Abstract

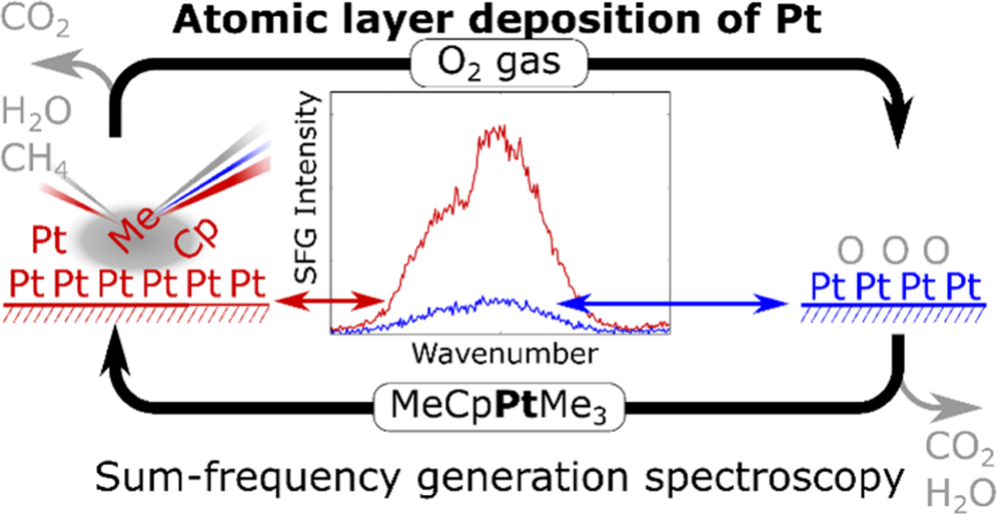

A detailed understanding of the growth of noble metals by atomic layer deposition (ALD) is key for various applications of these materials in catalysis and nanoelectronics. The Pt ALD process using MeCpPtMe_3_ and O_2_ gas as reactants serves as a model system for the ALD processes of noble metals in general. The surface chemistry of this process was studied by *in situ* vibrational broadband sum-frequency generation (BB-SFG) spectroscopy, and the results are placed in the context of a literature overview of the reaction mechanism. The BB-SFG experiments provided direct evidence for the presence of CH_3_ groups on the Pt surface after precursor chemisorption at 250 °C. Strong evidence was found for the presence of a C=C containing complex (*e.g*., the form of Cp species) and for partial dehydrogenation of the surface species during the precursor half-cycle. The reaction kinetics of the precursor half-cycle were followed at 250 °C, showing that the C=C coverage saturated before the saturation of CH_3_. This complex behavior points to the competition of multiple surface reactions, also reflected in the temperature dependence of the reaction mechanism. The CH_3_ saturation coverage decreased significantly with temperature, while the C=C coverage remained constant after precursor chemisorption on the Pt surface for temperatures from 80 to 300 °C. These SFG results have resulted in a better understanding of the Pt ALD process and also highlight the surface chemistry during thin-film growth as a promising field of study for the BB-SFG community.

## 1 Introduction

Ultrathin films and nanoparticles of noble metals have a wide range of (potential) applications associated with their catalytic nature, chemical stability, and high work function.^1−6^ Atomic layer deposition (ALD) of noble metals is gaining increasing interest for the fabrication of such ultrathin films and nanoparticles.^7−9^ This is mainly motivated by the unique combination of properties that ALD offers, including precise control over film thickness, unparalleled conformality over complex 3D structures, and superior uniformity across large substrates. As such, the application fields of noble metal ALD include catalysis and nanoelectronics.^5,10−15^ Insight into the reaction mechanisms of noble metal ALD processes is essential to extend their operating conditions, to enable new applications, and allow for deposition on challenging substrates (*e.g*., powders or polymers). This can be illustrated by the development of low-temperature variants of noble metal ALD: once it was understood that the O_2_ coreactant was not reactive enough below 200 °C, the issue was mitigated by introducing more reactive coreactants such as O_3_ and O_2_ or H_2_ plasma steps.^11,13,16,17^ Using the more reactive coreactants resulted in the capability to deposit high-quality material even at room temperature.^13^ Similar mechanistic insights were needed to reliably deposit Ru,^18^ Pd,^5,19^ Pt-Ir alloys,^3,15^ and Pt coatings on nanoparticles.^7,9^ Fundamental insight into the reaction mechanisms also aids in understanding aspects such as nucleation and island growth, which are of interest for the controlled growth of nanoparticles.^9,17,20^

**Figure 1:**
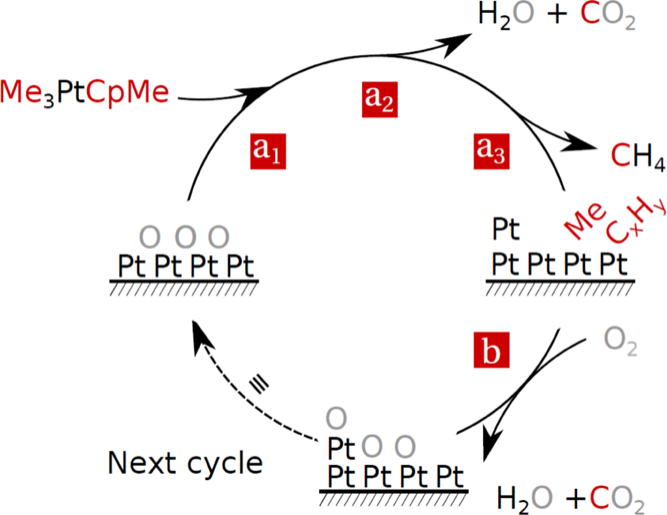
Current understanding of the Pt ALD reaction mechanism with MeCpPtMe_3_ and O_2_(g) as a precursor and a coreactant, respectively. At the beginning of the Pt half-cycle, there is a significant amount of atomic O adsorbed on the Pt surface. (**a**_**1**_) The MeCpPtMe_3_ precursor adsorbs on this surface. (**a**_**2**_) Some of the ligands are combusted to gaseous CO_2_ and H_2_O by the adsorbed atomic O. Other ligands either remain intact or undergo dehydrogenation to form adsorbed C_x_H_y_ and H. (**a**_**3**_) Some of the fragments recombine with adsorbed H on the surface to form CH_4_ gas species. In the coreactant half-cycle, O_2_ dissociates on the catalytic Pt surface and reacts with the remaining hydrocarbon groups forming gaseous CO_2_ and H_2_O. The surface is saturated with adsorbed atomic O at the end of the coreactant half-cycle.

The prototypical study case for noble metal ALD is the thermal Pt process using MeCpPtMe_3_ and O_2_ gas. While there are some differences between the platinum group metals in their capability to oxidize and their redox chemistry,^18,21^ the surface reactions catalyzed by the Pt surface capture the main mechanistic features of noble metal ALD. This means that the mechanistic insights from studying Pt ALD can be extended to other noble metals to a fair extent.^22^ Most of the mechanistic studies for Pt ALD have focused on either studying the process characteristics or the gas-phase reaction products. As a result, the reaction mechanism is understood in broad strokes, as is illustrated in Figure 1. In the precursor half-cycle, the MeCpPtMe_3_ precursor molecule adsorbs on oxygen-rich Pt (in the form of adsorbed O). This adsorption reaction deposits Pt and hydrocarbon species on the surface while releasing mainly CO_2_ and H_2_O as gas-phase reaction products.^23^ Precursor adsorption continues even when the adsorbed O is (largely) depleted but now producing CH_4_ as a gas-phase reaction product while consuming adsorbed H.^23^ Eventually, a carbonaceous layer forms, blocking the precursor absorption that is hypothesized as being the underlying mechanism of the self-limiting behavior of the precursor half-cycle. In the coreactant half-cycle, molecular oxygen adsorbs dissociatively and combusts the carbonaceous layer forming CO_2_ and H_2_O as gas-phase reaction products, removing the hydrocarbon groups and replenishing the O(ads) on the Pt surface.^23^ The understanding of the reaction mechanism of the ALD process so far is mainly based on indirect studies (probing, *e.g*., gas-phase reaction products) augmented by the works of Van Daele et al. and Geyer et al.^24,25^ Apart from these studies, insights from the field of surface science on noble metals are heavily relied on. Fundamental questions remain unanswered as a result of the lack of direct studies of surface chemistry. For example, the chemical structure of the precursor after chemisorption on the Pt surface is not known and it is unclear to which degree the precursor ligands are dehydrogenated on the catalytic Pt surface. To answer these and other questions and to get a better understanding of the reaction mechanism, direct measurements of the surface chemistry during the Pt ALD process (*i.e*., in situ studies) are needed.

In this work, the surface chemistry during Pt ALD will be studied in more detail with broadband sum-frequency generation (BB-SFG) spectroscopy directly probing the vibrational fingerprint of the surface species. BB-SFG spectroscopy is a nonlinear optical technique with submonolayer sensitivity and inherent surface selectivity. SFG is the state-of-the-art diagnostic used in surface science to study systems such as solid–liquid interfaces and buried polymer interfaces.^26−28^ Apart from our previous work, so far, there have been no dedicated SFG studies reporting on the surface chemistry during ALD. This work will highlight the promise SFG holds to study surface chemistry during ALD and thin-film growth in general, as is also recently illustrated by the related work studying CO on ALD-grown catalysts using XPS and SFG.^29^ With BB-SFG spectroscopy, the functional groups present on the surface during ALD can be identified and the (relative) density of surface groups can be followed *in situ*, as is illustrated by our earlier work monitoring the density of CH_3_, OH, and Si–H groups during ALD of Al_2_O_3_.^30−32^ Identification of the hydrocarbon species on the surface of the precursor is of particular interest for the Pt ALD process and, therefore, the CH stretch region of the IR spectrum was probed with BB-SFG spectroscopy. Apart from identifying surface groups, the reaction kinetics during the precursor half-cycle and the influence of temperature on the surface chemistry were also studied.

## 2 Experimental Section

### Sum-Frequency Generation

The nonlinear optical process of sum-frequency generation (SFG) is well suited to study surface chemistry by exploiting several properties of second-order optical processes. When a surface with surface chemical groups is exposed to two very intense light beams, the electric fields of the two beams interact via the polarization of the material. The two driving beams with photon energy ℏω_1_ and ℏω_2_ induce a mixed polarization *P⃗*(ω_1_ + ω_2_) distinct from *P⃗*(ω_1_) and *P⃗*(ω_2_) describing linear optics. The induced nonlinear polarization in turn radiates light with photon energy *ℏ*ω_*sfg*_ = *ℏ*ω_1_ + *ℏ*ω_2_.^33^ The relation between the polarization of the matter *P⃗* associated with the SFG beam and the strength of the electric fields of the two driving beams  and  is given as follows
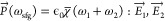
1where the χ̅ tensor describes the second-order susceptibility of the matter, which is a function of both ω_1_ and ω_2_ amongst other factors. From symmetry arguments, it can be deduced that the χ̅ tensor has to be zero in the bulk of a centrosymmetric material and no SFG signal can be generated.^33^ This implies that SFG does not occur in the bulk of amorphous materials and gasses nor in a large set of crystalline materials, including *c*-Si. At a surface, SFG is always allowed due to the lack of inversion symmetry.^33^ This makes SFG spectroscopy inherently surface-selective toward surface groups on most substrates and also rules out gas-phase contributions in virtually all cases.

### Vibrational

SFG spectroscopy is of particular interest for studying surface chemistry as it can probe the transitions between vibrational modes of the surface groups. Transitions between these vibrational states can cause a resonant enhancement of the SFG processes when one of the driving beams has photon energy matching the transition energy. Basically, the spectral shape of the resonance is reflected in the spectral shape of χ̅. Typical vibrational transitions have energy matching photons in the mid-IR part of the spectrum, dictating the choice of the photon energy for one of the two beams. For the other beam, visible light (∼800 nm) is typically chosen, and consequently, the resulting SFG photons are also situated in the visible part of the spectrum, which allows the use of highly sensitive detection schemes.

In *broadband* vibrational sum-frequency generation (BB-SFG), a broadband mid-IR pulse of femtosecond duration is mixed with a spectrally narrow visible pulse (typically picosecond). This effectively probes a region of the IR spectrum (∼200 around 3000 cm^–1^ for ∼90 fs IR pulses) at once without having to change the central wavelength of the mid-IR laser. The vibrational information is now contained in the spectral shape of the visible SFG signal. Assuming a spectrally narrow visible beam *I*_vis_ and a broadband IR beam *I*_ir_ (ω_ir_) (omitting the ω term for the visible beam emphasizing its discrete energy), the intensity *I*(ω_sfg_) of the detected SFG signal can be written as follows
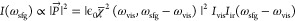
2

This means that both the spectral shape of χ̅ and that of the IR beam *I*_ir_ determine the shape of the final BB-SFG spectrum.

**Figure 2:**
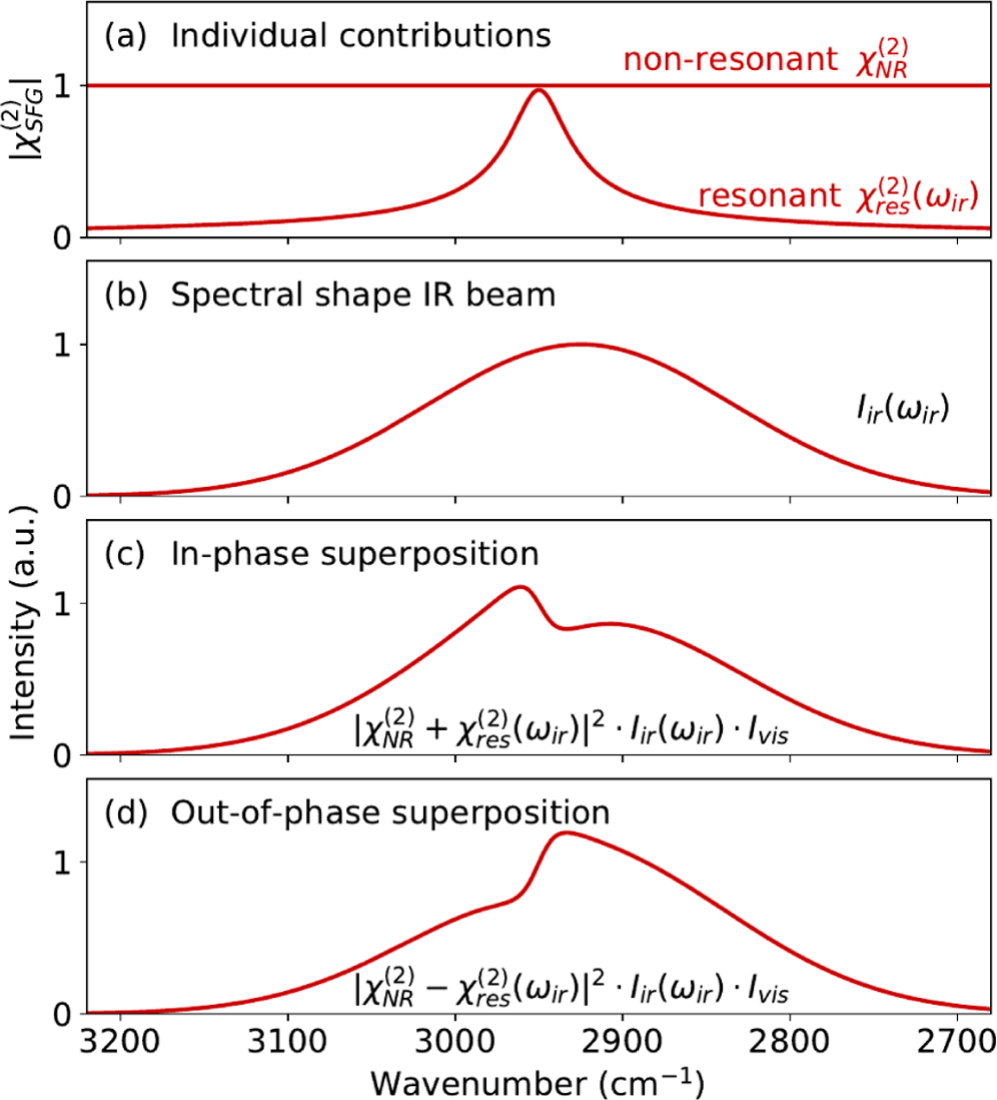
Shape of the BB-SFG response is affected by: (a) the spectral shape of *χ*, depending on the nature of the contribution, either being resonant (e.g., a C–H stretch) or nonresonant. (b) Spectral shape of the mid-IR beam used in the BB-SFG experiments. (c, d) Resulting BB-SFG response of an (c) in-phase and an (d) out-of-phase superposition of a resonant and a nonresonant contribution.

For the experiments in this work focusing on the surface groups on a noble metal, the BB-SFG spectra showed a coherent superposition of a nonresonant and a resonant contribution in χ̅. Both cases can be described using eq 2 with a different form of the second-order susceptibility for each. A nonresonant contribution has an amplitude and phase that do not vary with wavelength, so χ̅ can be represented as a single complex number. Conversely, a resonant contribution is described by a χ̅ strongly varying with photon energy, having an amplitude that peaks at a resonant frequency ω_res_ and its phase increases by π rad over the resonance. The spectral shape of a resonant contribution in the SFG spectra is described as follows^33^
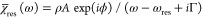
3where ρ is the density of the surface group associated with the resonance, *A* is the cross-section of the contribution (scaling approximately as the IR absorption cross-section multiplied with the Raman cross-section^26,33^), ϕ is the phase factor of the resonance, and Γ is the spectral broadening of the resonance. The tensorial nature of χ̅ was omitted for brevity. Note that the phase of the resonant signal varies with ω and is given by arg(χ̅) = ϕ – arg(ω – ω_res_ + *i*Γ), which is not equal to the phase factor ϕ alone. Figure 2a shows an example of the spectral shape χ̅ of both the resonant and nonresonant contributions. The shape of the mid-IR beam *I*_ir_ is a property of the laser system, and a representative shape can be seen in Figure 2b. In the case that both the resonant and nonresonant contributions are present, the combined response is a coherent (phase related) superposition of the two contributions multiplied with the spectral shape of the IR beam *I*_ir_ (ω_ir_). Therefore, the difference in phase between the two contributions influences the superposition. The two cases are illustrated in Figure 2, showing the *in-phase* superposition (ϕ = 0) in panel (c) and the out-of-phase superposition (ϕ∼π) in panel (d). These two cases were observed in the experiments (see Figures 4–7, for example).

To quantify the surface coverage ρ of a surface group causing a resonance in the SFG response, the measured BB-SFG spectra consisting of multiple contributions have to be deconvoluted. This allows, for example, to follow the (relative) surface coverages during ALD. The spectra were modeled using eqs 2 and 3, where the total response was given by χ̅(ω) = χ̅NR + χ̅_res_ (ω), with χ̅_NR_ being a real number. The shape of the mid-IR beam was determined by fitting a spectrum that only contained a nonresonant contribution, *e.g*., a clean surface. The central position, broadening, and phase of the resonant contribution were determined from a spectrum with a strong resonant component, and these parameters were fixed in subsequent fits. Then, the remaining spectra were fitted using a least-squares algorithm, varying the amplitude of the nonresonant and resonant contributions.

### ALD Setup

All experiments were performed in a home-built ALD chamber. The ALD chamber was equipped with two turbo-molecular pumps reaching a base pressure below 1 × 10^–6^ mbar. The MeCpPtMe_3_ precursor (Sigma-Aldrich 98% purity) was vapor drawn with a precursor temperature of 70 °C and a line temperature of 80 °C. The flow of O_2_ gas was regulated by a needle valve, and both the precursor and the coreactant were dosed using ALD valves driven by computer-controlled electronic relays. For most experiments, the relay was activated for 20 ms, which opened the ALD valve for the same duration. In some experiments, the relay was activated for only 6 ms to admit just a small amount of precursor into the ALD chamber. If the relay is activated for a duration approaching the relay release and bounce time (better than 5 ms and 3 ms, respectively), the exact pulse duration is not known, but tests showed that the duration is constant and repeatable. During the ALD cycles, the chamber was continuously pumped, with pressure varying between base pressure and ∼1 × 10^−^^3^ mbar (during the O_2_(g) pulse). The reactor walls were heated to 80 °C, and the Si substrate was heated radiatively with a Boralectric heating element. A thermocouple was glued to the back side of the sample with thermal paste. The sample temperature itself was computer-controlled by modulating the power dissipation in the heating element with the measured sample temperature as an input. This method of temperature control ensures a high accuracy (within 10 °C), which is important as the temperature has a significant effect on the Pt ALD process.^23,34^ The substrate was situated such that it can be studied with both *in situ* spectroscopic ellipsometry (SE) and *in situ* BB-SFG spectroscopy. For the *in situ* SE measurements, a J.A. Woollam Co. M2000U with a NIR extension (0.75–5.0 eV) was used.

The BB-SFG setup was home built and consisted of a 90 fs solid-state laser system to generate the visible 795 nm beam (Spectra-Physics Spitfire) and the tunable mid-IR beam operating around 3 μm (Spectra-Physics TOPAS-C). The BB-SFG signal was detected with a liquid nitrogen-cooled back-thinned CCD camera (Princeton Instruments Spec-10). In the ALD chamber, different Si substrates could be mounted, which in this case entailed substrates with a Pt or a SiO_2_ film on top. To study the surface chemistry on the Pt surfaces, *p*-polarized visible and mid-IR light was used to drive the SFG process. The *p*-polarized component of the SFG light was selected for detection using a polarizer, denoted by the upper-case letter *P*. The total polarization combination in the experiment is denoted as the *Ppp* polarization combination (*i.e*., going from high to low photon energy). The *Ppp* polarization combination typically results in the strongest signals for the surface group such as CH_3_ on Pt due to the metallic nature of the film. For experiments on the SiO_2_ surface, the *Ssp* polarization combination was used since it is known to yield the strongest signals for CH_3_ groups.^27^ A more detailed description of the experimental setup is given in our earlier work.^30,32^

### Sample Preparation

Both the BB-SFG and SE experiments were performed on two distinct surfaces: a Pt surface of a closed ALD-grown Pt film and the surface of a SiO_2_ film, both on a 2 inch Si(100) wafer. Because of the differences in the analysis techniques, slightly different samples were required to achieve the best sensitivity.

For the SE measurements on the SiO_2_ surface, a 350 nm SiO_2_ film was grown on top of the substrate by plasma-enhanced chemical vapor deposition (PE-CVD). This specific thickness was chosen for the best optical contrast for the SE measurement. For the experiments on the Pt surface, a second Si/SiO_2_ sample was prepared with the same procedure, after which a ∼30 nm Pt film was deposited on top of the SiO_2_ film.

For the BB-SFG experiments on the SiO_2_ surface, a ∼90 nm SiO_2_ film was grown on top of the substrate with PE-CVD. This specific SiO_2_ thickness yields the strongest SFG signals for experiments on a Si substrate. For the experiments on the Pt surface, the SiO_2_ layer was omitted and a thick Pt film was directly grown on top of the Si substrate. No optical enhancement of the SFG signal could be gained by adding a SiO_2_ layer in between the substrate and the Pt film.

## 3 Results and Discussion

### Characterization of the ALD Process with *In Situ* Spectroscopic Ellipsometry

**Figure 3:**
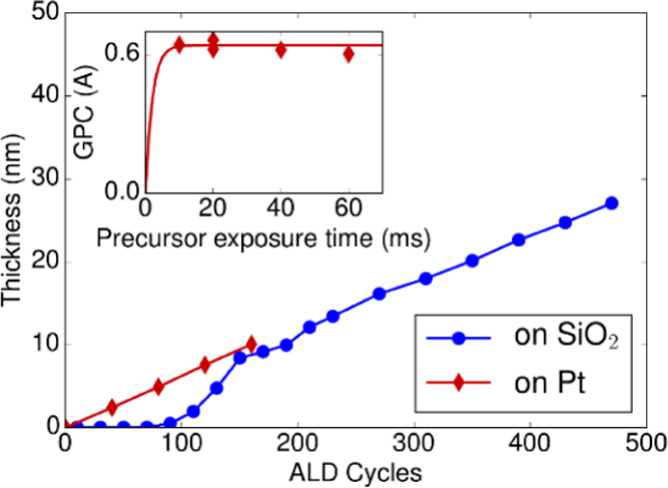
Thickness of an ALD-grown Pt film at 250 °C as a function of the number of ALD cycles for two different starting surfaces (Pt and SiO_2_) measured with *in situ* SE. For simplicity, the thickness of the initial Pt film is subtracted for the growth on Pt. The inset shows the saturation of the GPC of the precursor half-cycle for ALD in the steady-growth regime.

The ALD process was characterized at 250 °C, monitoring the Pt film thickness using *in situ* SE on both Pt and SiO_2_ surfaces, as shown in Figure 3. On the Pt surface, immediate growth was observed with a growth-per-cycle (GPC) of 0.06 ± 0.01 nm. The growth on the SiO_2_ surface showed a nucleation delay of ∼100 ALD cycles, after which the steady-growth regime was reached. These results are in line with the observations reported in the literature.^16,34^

The saturation behavior of the process in the steady-growth regime was also confirmed by monitoring the GPC while varying either the precursor or coreactant exposure during ALD on a thick Pt film. Saturation was observed for a precursor exposure of >10 ms (see the inset in Figure 3) and a coreactant exposure of >20 ms (not shown). A pump step in the order of ∼5 s was sufficient to purge away reaction products for either half-cycle.^18,23,34,35^ However, a longer pump step of 45 s and 30 s was used for the precursor and coreactant half-cycle, respectively. This timing resembles the experimental conditions used for the collection of the BB-SFG spectra.

### BB-SFG Spectra per Half-Cycle on Pt

**Figure 4:**
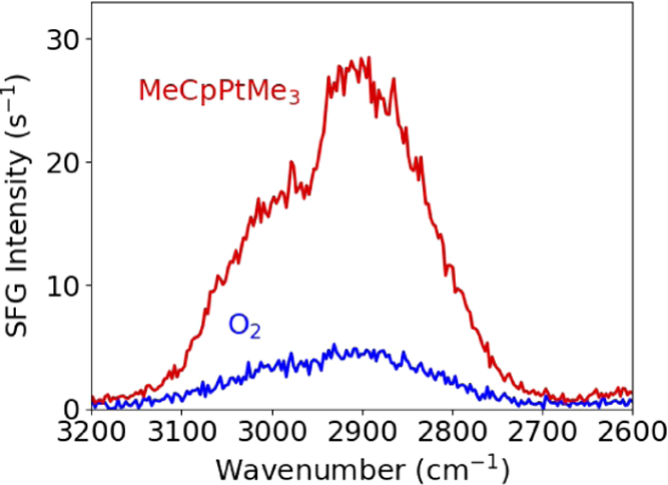
BB-SFG spectra of the C–H stretch region recorded at the end of the precursor (MeCpPtMe_3_) and coreactant (O_2_) half-cycles during ALD at 250 °C on a Pt surface. Dosing the precursor resulted in an increase in the broad nonresonant feature with an out-of-phase resonant feature at 2950 cm^–1^ superimposed on it.

To identify the surface species present during ALD at 250 °C, the C–H stretch region around 3000 cm^–1^ was probed with BB-SFG spectroscopy on a thick Pt film. Before recording the spectra, 5 Pt ALD cycles were performed to ensure that ALD has reached the steady-growth regime. Subsequently, the precursor was dosed, the reactor was pumped down, and a BB-SFG spectrum was recorded. The same procedure was used for the coreactant half-cycle, and the results are shown in Figure 4. The BB-SFG spectrum recorded after the O_2_ half-cycle shows a single broad feature. The shape of this feature is typical for a nonresonant contribution probed with BB-SFG spectroscopy, and it reflects the spectral shape of the mid-IR beam used in the experiment. To some extent, all metals have a nonresonant response and, therefore, this nonresonant contribution was assigned to the (thick) Pt film itself.^33^ In the BB-SFG spectrum recorded after the precursor half-cycle, an increase in the nonresonant contribution was observed together with the appearance of a small “dip” in the broad nonresonant feature. The overall shape is characteristic for the out-of-phase superposition of a resonant and a nonresonant contribution, see Figure 2d compared to Figure 4. The spectrum recorded after the precursor half-cycle was fitted and the spectral position of the resonant feature was found to be 2950 cm^–1^. This corresponds to the C–H stretch mode of both the −CH_3_ groups of the MeCp ligand and adsorbed CH_3_ groups on a Pt surface.^36,37^ Because the C–H stretch mode of both types of CH_3_ groups overlaps, it was not possible to differentiate between them and both these groups will be referred to as CH_3_ from this point onward. The C–H stretch mode of the CH_1_ on the cyclopentadienyl ring (C_5_H_4_) was not observed. In IR absorption spectroscopy, this mode is observed at ∼3100 cm^–1^ in, for example, gas-phase cyclopentane, cyclopentene, and cyclopentadiene.^38,39^ The absence of this contribution in the SFG spectra can be explained by the largely (centro)symmetric arrangement of the C–H groups on the Cp ring (strongly), reducing their SFG response.^36,40^ Finally, the change in the nonresonant contribution as shown in Figure 4, i.e., the large increase caused by the precursor exposure, was assigned to C=C bonds deposited on the surface during the precursor half-cycle. This assignment will be justified in the next section, and further verification will be shown. To summarize, three contributions have been identified in the BB-SFG spectra: a constant nonresonant contribution from the Pt film, a varying nonresonant contribution related to C=C bonds, and a resonant contribution from the CH stretch of CH_3_ groups.

From the spectra in Figure 4, the following conclusions about the surface chemistry of the Pt ALD at 250 °C can be drawn. (i) Not all precursor ligands undergo dehydrogenation on the surface; a significant number of CH_3_ groups remain on the surface as CH_3_ groups are attached to either the Pt surface or to the Cp ring. (ii) During the precursor half-cycle, both CH_3_ groups and C=C groups appear on the surface, and these groups are removed in the subsequent coreactant half-cycle.

### Origin of the Changing Nonresonant Contribution

Two different types of nonresonant signals were encountered: the signal of a clean Pt surface after the coreactant half-cycle and the change in this signal after the precursor exposure. The interpretation of the nonresonant signal after the coreactant half-cycle was already attributed to the Pt metal itself. The most likely causes of the *change* in the nonresonant contribution are (1) a change in the Pt surface, such as a reduction of the surface by the precursor (reverted back to its original state by subsequent oxidation). (2) A precursor ligand or fragment, which is added to the surface during precursor exposure (removed by the coreactant step). Note that a change in the thickness of the Pt film due to film growth by ALD can already be ruled out: the probing depth of BB-SFG (<4 nm) is much smaller than the Pt film thickness (>30 nm) used in the experiments.

**Figure 5:**
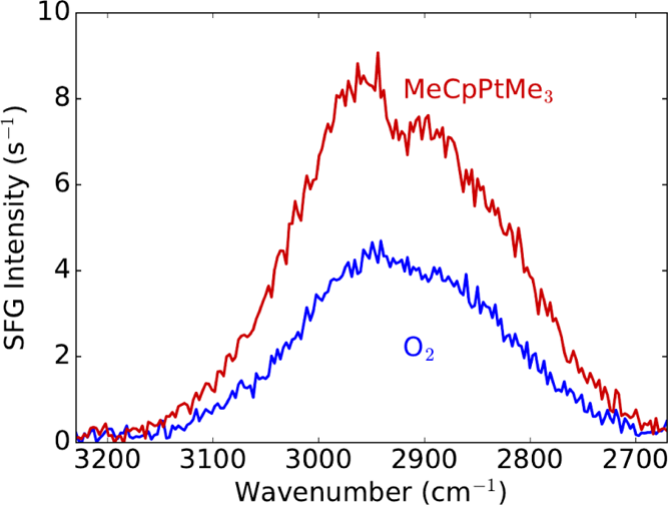
BB-SFG spectra of a SiO_2_ surface at 80 °C before and after exposure to the Pt precursor now using the *Ssp* polarization combination. The increase of the nonresonant contribution (broad feature) and the appearance of the resonant feature (shoulder) occur due to precursor exposure.

To test if the change in the nonresonant contribution is related to the precursor molecule, the ALD process was also studied on a SiO_2_ surface. The SFG response of the Si/SiO_2_ substrate is not expected to change during the ALD cycle: the SiO_2_ film of the Si/SiO_2_ substrate does not yield a measurable nonresonant contribution. The underlying Si, which does yield a nonresonant SFG signal, is shielded from the reactor environment by the thick SiO_2_ film. Figure 5 shows BB-SFG spectra recorded before and after precursor exposure of the Si/SiO_2_ substrate. Similar to the observations on the Pt surface, dosing the precursor resulted in an increase in the nonresonant contribution. The observation of a similar change of the nonresonant contribution on both the Pt and SiO_2_ surfaces points toward (a part of) the precursor as the origin of this signal. Moreover, instead of a dip, now a small shoulder appeared at the same position on the Si/SiO_2_ substrate. This different shape is expected given the very different linear optic properties of the two samples and the different polarizations (*Ssp* versus *Ppp*) of the beams with their respective Fresnel coefficients; see the SI for further details. This difference results in an out-of-phase superposition of the resonant and nonresonant contributions for the Pt sample and an in-phase superposition for the SiO_2_ sample; see also Figure 2c,d. The CH_3_ groups present on the surface result in a shoulder on the SiO_2_ surface and a dip on the Pt. The common origin of this resonant contribution is reflected in the two features having the spectral position and width, and the difference in shape is purely the result of the phase difference in the optical system.

**Figure 6:**
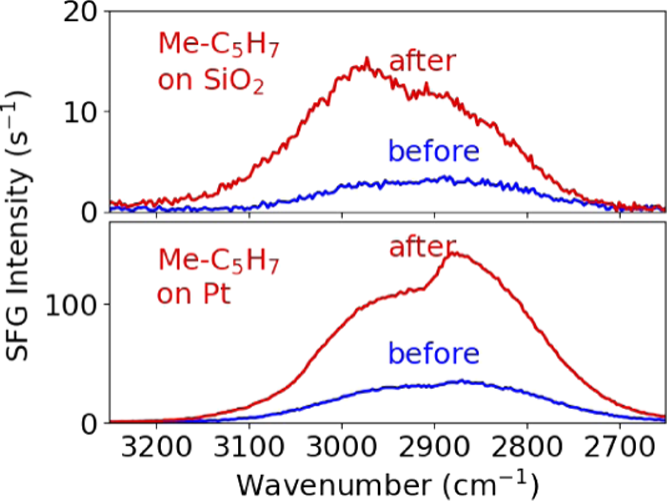
Comparison of the BB-SFG spectra probing the C–H stretch region on a SiO_2_ and a Pt substrate at 80 °C “before” and “after” the surface is exposed to Me-C_5_H_7_. Both spectra are very similar to those obtained with the Pt precursor, MeCpPtMe_3_, on both Pt and SiO_2_, as can be seen in Figures 3 and 4.

The methylcyclopentadienyl (C_5_H_4_CH_3_^**-**^) ligand of the precursor warrants further investigation as the cause of the nonresonant contribution. The MeCp ligand is added to the Pt surface in the precursor half-cycle and removed in the coreactant half-cycle, in line with the appearance and disappearance of the nonresonant contribution on both the Pt and SiO_2_ surfaces. To test if the change in the nonresonant signal is caused by the MeCp ligand, the state of the surface after precursor chemisorption can be mimicked. Ideally, one would dose the MeCp ligand of the precursor onto the surface. However, the neutral form of the ligand, *i.e*., methylcyclopentadiene (C_5_H_5_CH_3_), was not suitable for this purpose since it only exists as a dimer. On the other hand, 1-methylcyclopentene (C_5_H_7_CH_3_) is stable in the gas phase and is quite similar to methylcyclopentadienyl (it has 3 additional H atoms resulting in only 1 instead of 2 C=C bonds). Furthermore, it adsorbs onto the SiO_2_ and Pt surfaces at temperatures that could be achieved in the ALD reactor used.^41^ From this point on, the methylcyclopentene molecule will be referred to as “Me-C_5_H_7_” to differentiate it from the MeCp ligand of the precursor. The Me-C_5_H_7_ molecule was dosed onto the Pt and SiO_2_ surfaces at 80 °C (the lowest possible temperature given the wall temperature). Figure 6 shows the BB-SFG spectra recorded before and after the two surfaces were exposed to the Me-C_5_H_7_ molecule. On both surfaces, an increase in the nonresonant contribution was observed after exposing the surface to the Me-C_5_H_7_ molecule. At the same time, the dip (shoulder) related to the Me-C_5_H_7_ groups appeared on both surfaces. Again, the resonant contribution is in-phase with the nonresonant contribution on the SiO_2_ surface and out-of-phase on the Pt surface, mirroring the modeled spectra shown in Figure 2. The similarity of the results for the MeCpPtMe_3_ precursor and the Me-C_5_H_7_ molecule on both surfaces strongly suggests that the cause of the changing nonresonant background is due to the MeCp ligand of the precursor. The MeCp ligand consists of two parts: the CH_3_ group and the Cp ring. It is not expected that the CH_3_ groups (present in both the precursor and the Me-C_5_H_7_ molecule) are the cause of the change of the nonresonant contribution. Such an effect was not observed in, for example, thermal ALD of Al_2_O_3_ using Al(CH_3_)_3_ and H_2_O.^31^ This leaves the Cp ring as the likely origin of the change in the nonresonant contribution. Irrespective of whether the ring is intact or not on the (catalytic) Pt surface, in both cases, unsaturated carbon–carbon bonds are present on the surface that are known for their large nonlinear response.^33^ This has led us to assign the changing part of the nonresonant contribution to C=C bonds present in the MeCpPtMe_3_ precursor and the Me-C_5_H_7_ molecule. Hence, the changing nonresonant contribution could be seen as an indication that groups with C=C bonds are present on the surface but not necessarily in the form of Cp.

An additional check was performed using the Me-C_5_H_7_ molecule to confirm the nonresonant nature of the C=C contribution. If the changing part of the broad feature is a truly nonresonant contribution, the same behavior should be observed over a wide spectral range. The BB-SFG response around 2700 cm^–1^ was recorded before and after exposing the Pt surface to the Me-C_5_H_7_ molecule; see also Figure S1 in the supporting information. Apart from the resonant CH_3_ contribution, which is now outside the probed spectral range, the same behavior was observed around 2700 cm^–1^ as was observed around 3000 cm^–1^: An increase in the nonresonant contribution was evident after dosing the Me-C_5_H_7_ molecule on the Pt surface. This means that the nonresonant contribution is present from <2650 to >3100 cm^–1^, further pointing toward a nonresonant nature of this signal.

### Surface Termination as a Function of Temperature

**Figure 7:**
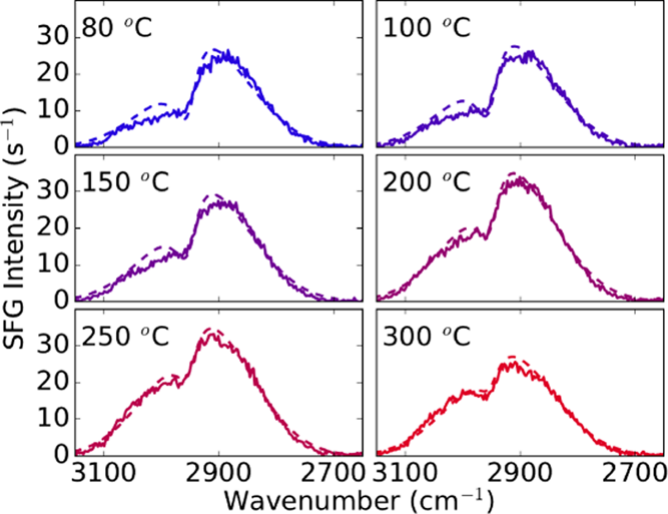
BB-SFG spectra obtained after dosing the precursor on a thick Pt film for substrate temperatures ranging from 80 to 300 °C. The dashed lines are fits to the data.

**Figure 8:**
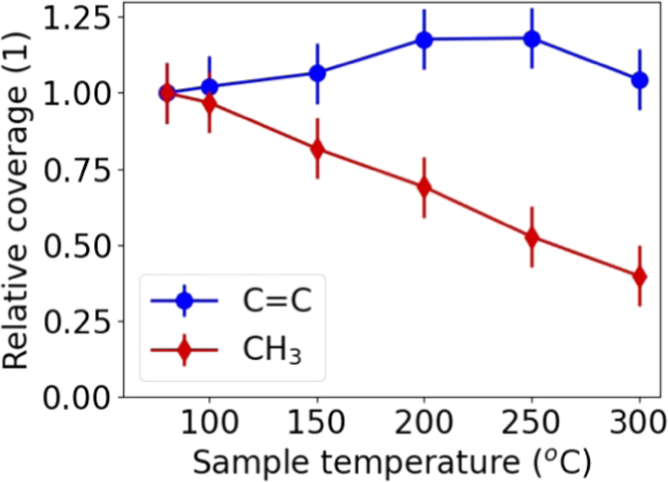
Trend in the relative coverage of the CH_3_ (either bonded to Pt or to the Cp ring) and C=C groups with temperature. This data was obtained from a fit of the BB-SFG spectra in Figure 7 and normalized to the coverage at the lowest temperature.

The impact of temperature on the surface coverage of the CH_3_ and C=C groups after the precursor half-cycle was studied with BB-SFG spectroscopy for temperatures ranging between 80 and 300 °C. For each temperature, the Pt surface was cleaned at a high temperature (∼300 °C) with a long O_2_(g) exposure. The sample was then allowed to cool down to the appropriate temperature, exposed to the precursor, and a BB-SFG spectrum was recorded. Figure 7 shows the BB-SFG spectra recorded after the precursor exposure at different temperatures. All of the spectra show the typical shape indicative of precursor adsorption with the coherent superposition of a resonant signal related to the CH_3_ groups and the nonresonant signal related to the C=C groups. The strength of the CH_3_ signal decreases with temperature, while the C=C signal remains relatively constant. Figure 8 shows the normalized CH_3_ and C=C coverage as a function of temperature obtained from the fit to the spectra in Figure 7. From 80 °C up to 300 °C, a monotonic decrease in CH_3_ coverage was observed, while the C=C coverage was constant within experimental accuracy. This suggests that the Cp ring of the precursor remains on the surface at all temperatures, and although they might undergo structural changes, the Cp ring is not lost by, *e.g*., combustion reactions. The CH_3_ groups on the surface originating from the precursor are lost, which can be explained by a combination of combustion and (de)hydrogenation reactions. However, since the amount of O(ads) available for combustion is expected to be relatively constant over this temperature range,^42−44^ the increasing loss of CH_3_ with temperature makes it plausible that (de)hydrogenation reactions contribute significantly to the loss of CH_3_ at ALD temperatures between 200 °C up to 300 °C.

### Reaction Kinetics during the Precursor Half-Cycle

**Figure 9:**
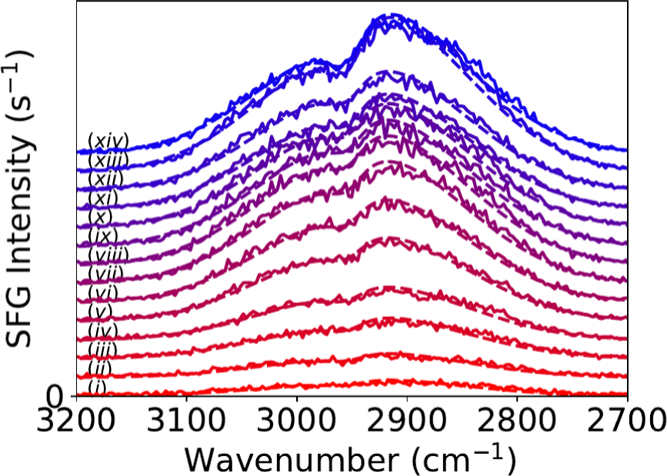
BB-SFG spectra of the C–H stretch region obtained for sequential precursor exposures revealing the reaction kinetics during ALD at 250 °C. Each spectrum has been offset vertically for clarity; the dashed lines are fits to the data.

**Figure 10:**
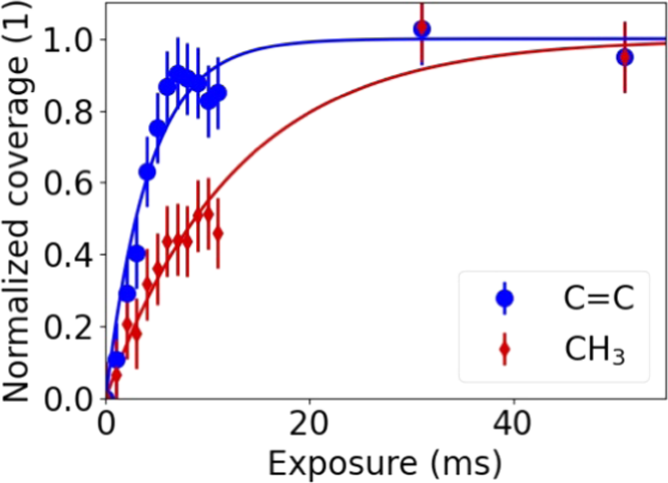
Trends in the relative coverage of the CH_3_ (either bonded to Pt or to the Cp ring) and C=C groups obtained from fits in Figure 9. The effective exposure time for the data points below 11 ms was estimated. The last two exposures had a duration of 20 ms. The solid lines represent a fit of the data with an exponential function with a single time constant.

The evolution of the surface chemistry during the precursor half-cycle was followed for ALD at 250 °C. The Pt surface was again prepared by performing five ALD cycles to ensure that the growth has settled into the steady-growth regime. After the O_2_ half-cycle of the fifth ALD cycle, a BB-SFG spectrum was recorded, resulting in spectrum (i) in Figure 9. Subsequently, multiple short exposures of precursor were performed by activating the electronic relay of the ALD valve for 6 ms (approaching the valve’s minimum response time), which admits a fraction of the standard amount of precursor into the ALD chamber. Using a standard pulse of precursor would not have yielded sufficient insight into the reaction kinetics since the SE measurements showed that this standard pulse is already sufficient to reach saturation. In total, 11 of these short exposures were performed, shown as spectra (ii) to (xii). After this series, two standard 20 ms exposures were performed. The first exposure of 20 ms (xiii) ensures saturation and the second exposure of 20 ms (xiv) provides an indication of repeatability of the measurement and confirms the saturation. Figure 9 shows the BB-SFG spectra recorded after each exposure. The typical spectral shape seen in the earlier experiments was again observed with both the resonant and nonresonant contributions increasing with precursor exposure. The effective dose of the short exposures was estimated to be equivalent to a ∼1 ms exposure judging from the number of pulses needed to reach saturation of the C=C signal. The spectra in Figure 9 were quantified by the fitting procedure discussed earlier, resulting in Figure 10. As can be seen in Figure 10, both the CH_3_ and C=C coverages increase gradually with precursor exposure. The C=C coverage reaches saturation after the first 11 exposures, with the coverage of the two long exposures indeed confirming that saturation has been reached; however, the CH_3_ coverage only reached saturation after the first long exposure. It is peculiar that the two contributions show a large difference in saturation behavior, and this points toward different reaction paths for C=C and CH_3_.

The trends in the CH_3_ and C=C coverage in Figure 10 were modeled assuming a single exponential function (1 – exp(−*t*/τ)), shown as the solid lines in the figure. Although the reaction kinetics are expected to be more complex than first-order, this approach still provides insight into the relative reactivity of either species. The time constants obtained from these fits were τ_CC_ = 4 ms for the C=C contribution and τ_CH3_ = 13 ms for the CH_3_ contribution. In this analysis, the dose time for the first 11 data points was taken to be ∼1 ms, and although the absolute dose time is not well known, the repeatability was excellent. As a result, the ratio of the two time constants τ_CC_/τCH_3_ is accurate and can be interpreted. It turns out that the C=C contribution reached saturation ∼3 times faster than the CH_3_ contribution. This faster buildup of C=C compared to CH_3_ could be explained by the quenching of CH_3_ loss paths with the increasing precursor dose. The Pt surface is O rich at the beginning of the precursor half-cycle, and most likely, the CH_3_ groups undergo combustion reactions, but at the same time, dehydrogenation of CH_3_ also occurs. The loss path resulting from the hydrogenation of CH_3_ to CH_4_ starts to contribute once sufficient H(ads) is present. All in all, this would lead to a significant loss of CH_3_ at the beginning of the precursor half-cycle. Near saturation of the precursor half-cycle, the combustion reactions are quenched due to the depletion of O(ads). The (de)hydrogenation is also quenched because the presence of hydrocarbon species makes the surface less reactive. The strongly quenched loss paths for CH_3_ but steady built-up of the C=C corresponds to a changing ratio of the deposited CH_3_ to C=C as a function of dose. The relatively greater loss of CH_3_ at the onset of the half-cycle might explain the initially more rapid built-up of C=C compared to CH_3_.

## 4 Discussion

In this section, the main insight into the reaction mechanism of Pt ALD from the BB-SFG experiments will be summarized. This is followed by a broader overview of the current understanding of the reaction mechanism based on ALD studies, surface science studies, and the insight from this work.

### Insights into the Reaction Mechanism Obtained by BB-SFG

The insights obtained in this work complement and extend our understanding of the reaction mechanism of Pt ALD using MeCpPtMe_3_ and O_2_ as reactants. The results presented in this work show that:

- after the precursor absorption, CH_3_ groups and surface species with a C=C bond (such as in the Cp ring) are present on the surface. These groups are removed by O_2_ exposure at 250 °C; see the BB-SFG spectra in Figure 4. The BB-SFG signal is proportional to the coverage and not to the change in coverage as is the case for, *e.g*., FTIR difference spectra, which rules out any persistent CH_3_ species after the O_2_ exposure as having been seen for other ALD processes.^31,45,46^
- Not all CH_3_ groups undergo complete dehydrogenation in the precursor half-cycle since Figures 4 and 8 show clear evidence of CH_3_ on the surface at the end of the precursor half-cycle.
- The temperature has a strong impact on the relative amounts of the surface species at the end of the precursor half-cycle: Figure 8 shows that the amount of C=C is fairly constant, while the CH_3_ coverage decreases with temperature.
- The temperature trend of the CH_3_ coverage in Figure 8 is also evidence for the occurrence of (de)hydrogenation reactions. The amount of O(ads) available for combustion reactions is reported to be constant over this temperature range leaving (de)hydrogenation as the main cause of the lower CH_3_ coverage at high temperatures.
- The BB-SFG spectra in Figure 8 show that the precursor absorbs on the Pt surface at temperatures ranging from 80 °C up to 300 °C. This indicates that precursor adsorption is not the limiting step for thermal ALD, *i.e*., precursor adsorption is not the cause of the lack of growth of Pt at temperatures below 200 °C. This is in-line with earlier insights from ALD with O_3_ and O_2_-plasma, which also shows growth at lower temperatures.
- The complexity of the reaction mechanism of the precursor half-cycle (consisting of multiple competing reactions) is reflected in the reaction kinetics, as can be seen in Figure 10, and simple first-order reaction kinetics can be ruled out based on this data. The CH_3_ buildup is approximately 3× slower than the C=C buildup.
- The reaction kinetics qualitatively show a more rapid buildup of C=C as compared to CH_3_. This points to a quenching of the loss paths of CH_3_, which are the (de)hydrogenation and oxidation reactions. At the start of the precursor exposure, a significant fraction of CH_3_ ligands is lost because of the combustion, hydrogenation, and dehydrogenation reactions. This results in the deposition of relatively few CH_3_ groups per C=C containing group. Near saturation, the reaction paths for CH_3_ loss are quenched, increasing the number of CH_3_ deposited precursor molecules.

### Overview of the Pt ALD Reaction Mechanism

Here, the current mechanistic understanding of the growth of Pt ALD using MeCpPtMe_3_ as a precursor and O_2_ as a coreactant will be summarized, focusing on the steady-growth regime (Pt ALD on a Pt surface) for temperatures at which ALD growth occurs (200–300 °C). The structure of this section is as follows. First, an overview of both half-cycles will be given without going into details or citing specific works. This is followed by a detailed discussion of each reaction step, referring to the direct and indirect evidence in the literature.

The precursor half-cycle starts out with a Pt surface that is oxygen rich. This O-rich Pt surface is exposed to the gas-phase Pt precursor MeCpPtMe_3_, which adsorbs on the surface, as shown in Figure 1. Upon absorption, Pt is deposited and some of the (fragmented) precursor ligands remain on the surface
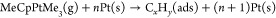
4with C_*x*_H_*y*_(ads) denoting the various hydrocarbons on the surface, such as CH_3_ groups and (fragments of) the Cp. Note that **reaction (4)** and the following reactions are not balanced in terms of constituent amounts because this level of detail of the surface chemistry is not available yet.

During the initial stage of the precursor half-cycle, the dominant gas-phase reaction product is CO_2_,^23^ most likely formed in a combustion-like reaction described by
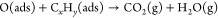
5Most studies do not explicitly report on the production of the H_2_O in **reaction (5)**, with some exceptions,^40^ because its presence is often difficult to detect unambiguously in a vacuum chamber under ALD(-like) conditions. The formation and role of the OH group have been studied by Elliott using density functional theory (DFT), yet experimental data is still lacking.^47^ The formation of CO_2_ and H_2_O leads to the depletion of the adsorbed O on the Pt, thereby eventually quenching the combustion reactions. As a result, a significant decrease in the production of CO_2_ is observed as the precursor half-cycle progresses.^23^

Gradually new reaction paths open up during the precursor half-cycle, mainly witnessed by the formation of increasing amounts of CH_4_, which eventually becomes the dominant gas-phase reaction product.^23^ This CH_4_ is most likely formed in a hydrogenation reaction of the C*_x_*H*_y_*(ads), mostly in the form of CH_3_ with H(ads) on the surface
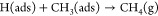
6Volatile higher hydrocarbons could also be formed but so far the observation of these species has not been reported for ALD. The H(ads) required for this reaction is most likely formed by dehydrogenation reactions of C_*x*_H_*y*_(ads) groups on the Pt surface^23,35^
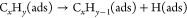
7Considering the overall trends (kinetics) of the precursor half-cycle, initially, the Pt surface is O rich, virtually no H(ads) is present, and hence mainly CO_2_ and H_2_O are formed. At high O coverage, **reaction (5)** dominates over **reaction (6)**. Once the O(ads) is depleted and sufficient H(ads) is present, the main gas-phase reaction product shifts from predominantly CO_2_ to nearly exclusively CH_4_, which remains the main gas-phase reaction product until the reactions self-terminate.^23^ The self-terminating nature of the precursor half-cycle is ascribed to the built-up of carbonaceous species on the surface.^24,35^

After this discussion of the precursor half-cycle in its entirety, each individual reaction step, *i.e*., **reaction (4)–(7)** and the self-limiting nature, will be addressed in more detail and supporting evidence for the earlier statements will be given. First, the evidence for each step reported in the ALD literature will be discussed, followed by the insights from related surface science experiments where applicable.

**Reaction (4)** describes the precursor adsorption on a Pt surface, and it has been demonstrated that a large fraction of the C of the precursor (79%) remains on the surface at the end of the precursor half-cycle. This was deduced in several quantitative analyses of the gas-phase reaction product of both half-cycles.^16,18,23,25,40^ The form of the C-containing species on the surface after precursor absorption is not fully clear. Geyer et al. used *in-operando* XPS to study the surface species during ALD. For the precursor half-cycle, they showed direct evidence for the deposition of Pt- and C-containing species on the surface, but it was not possible to pinpoint the exact chemical nature of the C species.^24^ In an extensive *in situ* FTIR study by Van Daele et al., the presence of precursor fragments on the surface in the form of C=C was confirmed and their work also showed evidence for CH_3_ groups on the surface.^25^ In the BB-SFG experiments presented in this work, both the C=C containing precursor fragments and CH_3_ were observed. At the beginning of the precursor half-cycle, about ∼0.3 monolayer of O(ads) is present on the Pt surface (not explicitly stated in **Reaction (4)**). This adsorbed O is not required for precursor absorption, as shown by Erkens et al. and others.^13,23,40^ However, the presence of O(ads) at the beginning of the precursor half-cycle allows for a (significantly) larger amount of precursor adsorption, while the O_2_ coreactant step is also needed for a sustainable ALD process.^35^ Overall, precursor adsorption results in the deposition of Pt atoms and leads to the presence of various hydrocarbon species on the surface, including CH_3_, unsaturated hydrocarbons (*i.e*., groups with a C=C bond) such as Cp, and other higher hydrocarbons.

**Reaction (5)** describes the combustion-like reactions that both the Me and MeCp precursor ligands can undergo in the presence of the O(ads) on the Pt surface. In ALD, this manifests itself as the production of CO_2_ and H_2_O as gas-phase reaction products with ample reports on the detection of the gas-phase species but with few reports directly probing the underlying surface chemistry leading to the production of CO_2_ and H_2_O.^16,18,23,40^ The surface chemistry of hydrocarbons on Pt surfaces (in the presence of O) has been studied extensively in the field of surface science. Combustion reactions, or more generally oxidation reactions, are known to occur on various O-rich Pt surfaces at ALD relevant temperatures for both Me groups and unsaturated cyclic hydrocarbons such as MeCp.^48−52^ For example, Marsh et al. studied the oxidation of benzene on a Pt(111) surface and demonstrated that above >300 K benzene is oxidized, forming CO_2_ and H_2_O. They observed additional combustion pathways opening up at >530 K in the presence of OH(ads).^51,52^ These oxidation reactions are likely to occur during ALD and would explain the formation of CO_2_ and H_2_O during the initial phase of the precursor step.

**Reaction (6)** describes the hydrogenation reactions of the CH_3_ precursor ligands on a Pt surface in the presence of H(ads). This reaction forms volatile species such as CH_4_ or other higher hydrocarbons such as C_2_H_6_. During ALD, the formation of CH_4_ as a gas-phase reaction product has been observed by QMS and gas-phase FTIR spectroscopy, especially in the latter part of the half-cycle.^18,23,40^ Higher hydrocarbons have not been observed; however, their formation is not ruled out because these species are more difficult to detect. In surface science, the hydrogenation of both CH_3_ and MeCp on Pt in the presence of H(ads) has been studied using a selection of hydrocarbons. For isolated CH_3_ groups on Pt(111) in the presence of H(ads), the formation of CH_4_ already starts at temperatures as low as 250 K,^53,54^ which suggests that the newly introduced CH_3_ groups on the surface from precursor adsorption will also rapidly lead to the formation of CH_4_ at ALD temperatures. The MeCp groups of the precursors (methylcyclopentene) have been shown to recombine with H(ads) and leave the surface as MeCp* (methyl cyclopentane) already occurring at a temperature of around 300 K.^55,56^ These hydrogenation reactions are the likely mechanism behind the observed gas-phase CH_4_ and higher hydrocarbons while also consuming H(ads) during the latter part of the precursor half-cycle

**Reaction (7)** describes the dehydrogenation reactions that the precursor ligands can undergo on a Pt surface. So far, only indirect evidence for dehydrogenation reactions during Pt ALD has been reported. Surface FTIR spectroscopy during ALD showed the presence of C=C bonds (already present in the precursor) and also the presence of C_x_H_y_ and Me. In this work, both the CH_3_ and C=C species are observed and their reaction kinetics reflect the complex interplay between the aforementioned oxidation, hydrogenation, and dehydrogenation reactions. In the field of surface science, the dehydrogenation of Me on Pt(111) has been studied by Fairbrother et al.^53^ They showed that Me groups either undergo dehydrogenation in the absence of H(ads) or recombine in the presence of H(ads) to form CH_4_ at temperatures relevant for ALD, and similar results were obtained by others on different facets of the Pt crystal.^53,54,57^ The dehydrogenation of MeCp, Cp, and Cp-like cyclical molecules such as benzene on Pt has also been studied. For example, dehydrogenation for MeCp on Pt(111) has been shown by Morales and Zaera to occur above 350 K.^58^ Similar studies for other unsaturated cyclic hydrocarbons have been performed, finding similar temperature thresholds for dehydrogenation.^56,59^ These dehydrogenation reactions do not directly show up during ALD (for example, as a gas-phase reaction product) but they do play a key role in the formation of CH_4_ as this reaction is the most likely source of H(ads) required for the hydrogenation reactions discussed earlier and could contribute to the self-limiting nature of the ALD reactions.

The mechanism responsible for the self-limiting nature of the precursor half-cycle is understood at a high level and is ascribed to the built-up of carbonaceous species on the surface, reducing its reactivity (*i.e*., a kind of poisoning). As is clear from the discussion so far, this is most likely a complex process consisting of many parallel and sequential reaction steps, so no (meaningful) reaction equation can be given. The presence of carbon on the surface at the end of the precursor half-cycle is indirectly (but irrefutably) proven by the production of CO_2_ in the coreactant half-cycle upon O_2_ gas exposure.^18,23,40^ The carbon is also observed more directly in the form of C=C in the SFG experiments in this work and also in the FTIR work by Van Daele et al. and in the XPS studies by Geyer et al. in the form of amorphous carbon.^24,25^ In surface science, carbon containing surface species are known to poison various gas-surface reactions of the Pt surface. Marsh and Somorjai showed that the presence of CO(ads) quenches the hydrogenation reactions of (cyclic) olefins.^60^ CO(ads) groups are indeed observed at the end of the precursor half-cycle during ALD by Van Daele et al. and others.^25,61^ Furthermore, the oxidation reactions of CH_3_ and cyclic hydrocarbons are poisoned by the presence of CO(ads) and OH surface groups; however, considering the scarcity of O(ads) at the end of the precursor half-cycle, oxidation reactions are most likely of less importance.^48,52^ In general, the formation of a carbonaceous layer—mainly consisting of unsaturated hydrocarbons and also referred to as coke—is known to form when a Pt surface is exposed to higher hydrocarbons, which reduces the reactivity of the surface.^62−64^ For ALD, a reduced reactivity of the surface due to the built-up of a carbonaceous layer is the most likely mechanism responsible for the self-limiting nature of the precursor half-cycle.

The coreactant half-cycle starts with a Pt surface covered with hydrocarbon species, which are exposed to gas-phase O_2_, as illustrated in Figure 1. Throughout the coreactant half-cycle, CO_2_ is reported as the gas-phase reaction product, described by **reaction (5)**, with H_2_O most likely also being formed but being difficult to detect, as mentioned before.^18,23,40^ The SFG spectra recorded after the O_2_ half-cycle in this work shows the removal of C=C species and the elimination of all CH_3_ from the surface. For a Pt surface covered by a coke layer formed from MeCp, it has been shown in the field of catalysis that O_2_ can remove this layer at temperatures starting around 200 °C but slightly higher temperatures have also been reported depending on the nature of the carbonaceous layer.^51,64^ Once the coke layer is (partially) removed, other reaction paths open up. For a Pt surface with adsorbed CO, the presence of O(ads) or gas-phase O_2_ both results in the formation of CO_2_ at temperatures relevant for ALD.^42^ For pristine polycrystalline Pt surfaces, dissociative adsorption of O_2_ is known to occur, driving combustion **reaction (2)** and replenishing the O(ads) needed for the next precursor half-cycle.^42,65^ The presence of O(ads) on the Pt surface at the end of the half-cycle was also directly observed by in-operando XPS measurements.^24^ The coreactant half-cycle reaches saturation when all of the hydrocarbons have been removed from the Pt surface and the O(ads) has been replenished.^23,35^

## 5 Conclusions

The reaction mechanism of Pt ALD using the reactants MeCpPtMe_3_ and O_2_ gas was investigated with *in situ* SE and *in situ* BB-SFG spectroscopy. The SE measurements confirmed typical ALD behavior, including the saturation of both half-cycles. The BB-SFG spectra showed direct evidence for the presence of CH_3_ on the surface at the end of the precursor half-cycle. From these observations, it can be concluded that not all precursor ligands undergo combustion or (de)hydrogenation reactions on the catalytic Pt surface. Carbon containing precursor fragments were also detected by BB-SFG, which were associated with surface species containing C=C, as pinpointed in a separate series of experiments absorbing various molecules on Pt and SiO_2_ surfaces. The reaction kinetics and the temperature dependence of the surface coverage of the CH_3_ and C=C containing species during the precursor half-cycle were studied with BB-SFG providing a more detailed picture of the reactions occurring during the precursor half-cycle. The observed reaction kinetics reflect the complexity of this reaction mechanism in line with the multiple competing reaction pathways. Evidence for (de)hydrogenation reactions was found from the temperature studies. For the O_2_ half-cycle, the results showed that both the CH_3_ and C=C groups are efficiently removed from the surface by the O_2_ exposure at 250 °C. To summarize, this work demonstrates the strength of BB-SFG spectroscopy for studying the surface chemistry and reaction kinetics during ALD on metallic films.

## Supporting Information Available

Additional spectra further support the assignment of the nonresonant contribution to unsaturated hydrocarbons; a full deconvolution of a BB-SFG spectrum into the resonant and nonresonant contribution; and a description of the modeling of the SFG response of the Pt and SiO_2_ surfaces (PDF)
