# Supplementary material for: Surface Chemistry during Atomic Layer Deposition of Pt Studied with Vibrational Sum-Frequency Generation
Source: J Phys Chem C Nanomater Interfaces. 2022 Jan 31;126(5):2463–74. doi: 10.1021/acs.jpcc.1c06947 (PMC8842249; doi:10.1021/acs.jpcc.1c06947)
Supplement: Supplementary file 1 — jp1c06947_si_001.pdf [file jp1c06947_si_001.pdf]

Surface Chemistry During Atomic-Layer Deposition of Pt Studied With Vibrational Sum-frequency Generation

V. Vandalon [1], A.J.M. Mackus [1], W.M.M. Kessels [1]
[1] Department of Applied Physics, Eindhoven University of Technology, 5600MB Eindhoven, The Netherlands

Supporting information

Additional evidence for the non-resonant nature related to the Cp ring.

The change in the amplitude in the non-resonant contribution in the BB-SFG spectra was attributed to C=C groups on the surface. To confirm the non-resonant nature of this contribution, it was verified that the increase in the non-resonant background upon Me-C_5H_7 exposure also occurred in a spectral region without resonant contributions. The region around ~2700 cm^{-1} was chosen for this purpose and the mid-IR beam tuned appropriately. A BB-SFG spectrum was acquired of the Pt surface before being exposed to Me-C_5H_7, but after having been cleaned by O_2. Figure S1 shows the BB-SFG response before and after Me-C_5H_7 exposure. Also in this case the non-resonant contribution increased upon dosing Me-C_5H_7 onto the surface, further validating the assignment of the changing part of the non-resonant contribution to C=C bonds in the Me-C_5H_7 and the MeCpPtMe molecules adsorbing on the surface.

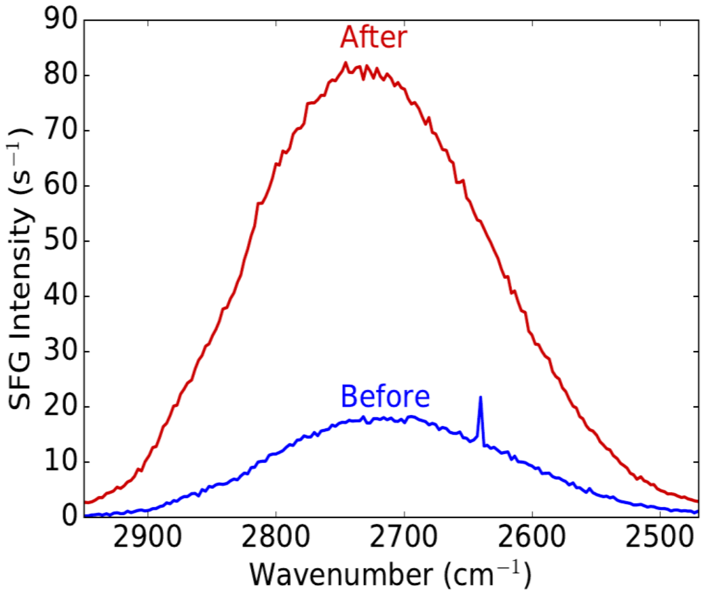

Figure S1: *BB-SFG response of the Pt surface before and after Me-C_5H_7 exposure in a part of the spectrum in which no resonant contributions are expected. Exposing the Pt surface to Me-C_5H_7 also results in a significant increase in the amplitude of the non-resonant SFG signal in this part of the IR spectrum. This demonstrates that the non-resonant contributions is spectrally broad and spans at least from 3100 cm^{-1} up to 2650 cm^{-1}. Note that the spectral shape reflects the spectral shape of the driving mid-IR beam which is also typical for a non-resonant contribution.*

Deconvolution of BB-SFG spectra

The spectra in Fig. 7 and Fig. 9 of the main text are deconvoluted to extract the amplitude of the resonant and non-resonant contributions. Figure S2 shows the deconvolution of the spectrum shown in Fig. 7 for 300 ^{o}C, now

also showing the individual contributions as shaded areas. The fit is a *coherent* superposition of the two contributions; plotting the individual contributions ($|A_{res}|^2$ and $|A_{non-res}|^2$) and therefore omits the interaction which typically leads to the heterodyne amplification of the weaker signal ($|A_{res}\, A_{non-res}|^2$ if $A_{res} < A_{non-res}$).

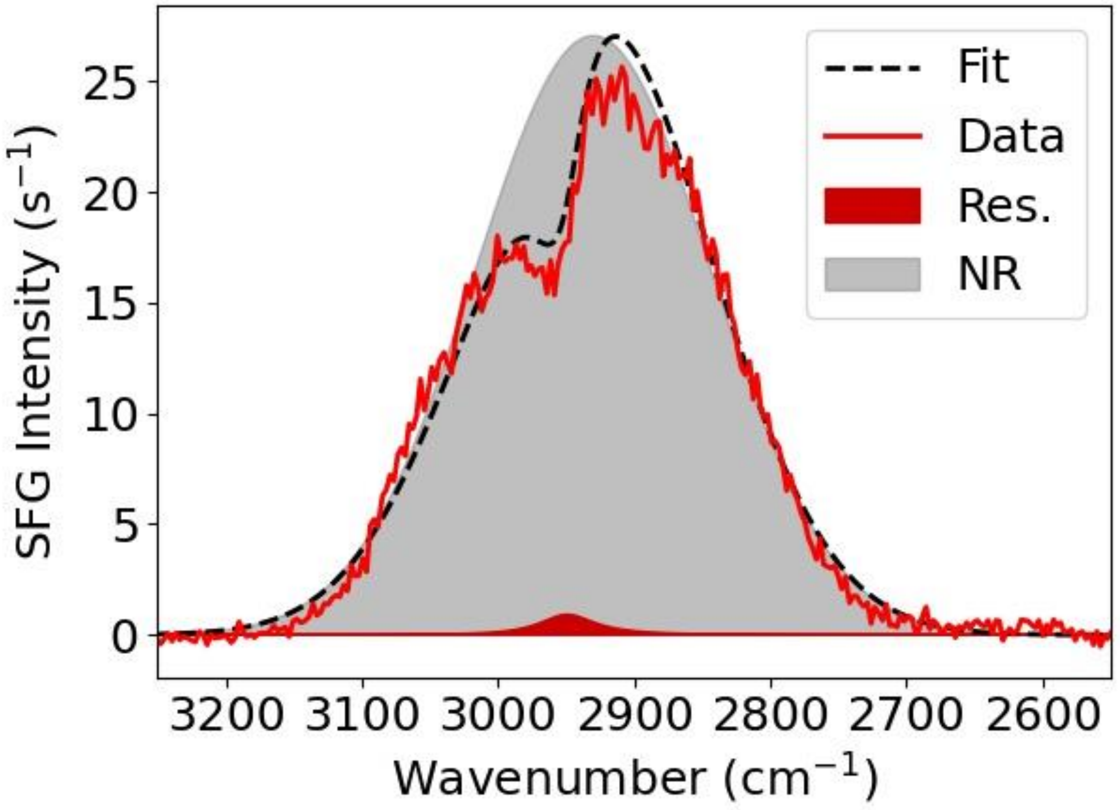

Figure S2: The deconvolution of the spectrum show in Fig. 7 in the main text for 300ºC showing the data, the fit envelope and its individual contributions.

Modeling of BB-SFG spectra

For the qualitative statement of the difference between the SFG spectra on Pt and SiO_2 with respect to phase, the phase of the SFG signal for the two cases was calculated. The sample with the SiO_2 surface consisted of a Si substrate with ~350 nm of SiO_2. The phase of this system was calculated taking into account their refractive index at 3300 nm, 800 nm, and 630 nm for the IR and visible beam impinging at a ~35 degrees angle on the surface. The well-known approach formulated by Sipe was used to model the propagation of the SFG light.[1] Similarly, the SFG on the Pt surface can be modeled yet internal reflections are absent since the light does not penetrate through the Pt layer. For the geometry used in our experiments a phase of 0.9π for the SiO_2 surface and 0.1π for the Pt surface were found. This phase is then used as the phase factor in Fig. 2 of the main text with a similar calculation for the Pt surface. Note however that for the deconvolution this analysis is not needed. These factors arising from the propagation of light through the sample are complex (with phase information) yet they are constant for each sample. Therefore they can be included scaling/normalization factor in the fit. They would needed when comparing SFG spectra on different samples in absolute terms.

References

1. Sipe, J. E. New Green-function formalism for surface optics. *J. Opt. Soc. Am. B* **4**, 481–489 (1987).
